# Layer-edge device of two-dimensional hybrid perovskites

**DOI:** 10.1038/s41467-018-07656-2

**Published:** 2018-12-05

**Authors:** Bin Cheng, Ting-You Li, Pai-Chun Wei, Jun Yin, Kang-Ting Ho, José Ramón Durán Retamal, Omar F. Mohammed, Jr-Hau He

**Affiliations:** 1 Computer, Electrical, and Mathematical Sciences and Engineering Division, King Abdullah University of Science and Technology, Thuwal, 23955-6900 Saudi Arabia; 2 Physical Sciences and Engineering Division, King Abdullah University of Science and Technology, Thuwal, 23955-6900 Saudi Arabia

## Abstract

Two dimensional layered organic-inorganic hybrid perovskites (2D perovskites) are potential candidates for next generation photovoltaic device. Especially, the out-of-plane surface perpendicular to the superlattice plane of 2D perovskites (layer-edge surface) has presented several exotic behaviors, such as layer-edge states which are found to be crucial for improving the efficiency of 2D perovskite solar cells. However, fundamental research on transport properties of layer-edge surface is still absent. In this report, we observe the electronic and opto-electronic behavior in layer-edge device of 2D perovskites. The dark and photo currents are demonstrated to strongly depend on the crystallographic orientation in layer-edge device, and such anisotropic properties, together with photo response, are related to the thickness of inorganic layers. Finally, due to the abundant hydroxyl groups, water molecules are easy to condense on the layer-edge surface, and the conductance is extremely sensitive to the humidity environment, indicating a potential application of humidity sensor.

## Abstract (web summary)

The edge of the thin sheets of two dimensional organic-inorganic hybrid perovskite crystals shows exotic physical properties that remain unexplored. Here Cheng et al. make electronic devices and study their transport properties, photoresponses and humid sensitivities which show large anisotropy.

## Introduction

Organic–inorganic hybrid perovskites MAPbX_3_ (MA = CH_3_NH_3_, X = Cl, Br, I; 3D perovskites for brief) have achieved a power conversion efficiency (PCE) of over 20%^1–5^ in photovoltaic solar cells and also are promising materials for fabricating light-emitting diodes^6,7^ and lasers^8,9^. Just like conventional semiconductor device^10,11^, the surfaces and interfaces have a key role in the electronic and opto-electronic performance in the hybrid perovskites based photovoltaic devices^12,13^. Especially in the two-dimensional-layered-organic–inorganic hybrid perovskites (2D perovskites), the out-of-plane surface perpendicular to the superlattice plane, which is named as layer-edge surface, has unique organic–inorganic staggered superlattice pattern, as a straightforward result of multiple quantum well structures. Because of the parasitic layer-edge states, such exotic layer-edge surface has a crucial role in dissociating the electron–hole pairs in the excitons into the free carriers and extend the lifetime of those carriers, leading to a largely improved efficiency in 2D perovskite based solar cells^14,15^. Moreover, a generic elastic model was supposed, demonstrating that the layer thickness-dependent electronic band structure of layer-edge surface in 2D perovskites comes from relaxation of mechanical strain in the mismatched interface^16^. Those impressive results presented fancy properties of the layer-edge surface in 2D perovskites, but several other important fundamental researches are still required, especially the electronic transport properties which is one of the key factors to determine the performance of opto-electronic devices.

Here, we succeed to grow centimeter-wide and millimeter-thick single crystalline 2D perovskites, and fabricate the metal–semiconductor-metal devices on the layer-edge surface in the direction parallel to the Pb–I plane (defined as layer-edge 0° device) and perpendicular to the Pb–I plane (defined as layer-edge 90° device), respectively. Transport measurements are performed in these layer-edge devices of 2D perovskites, showing highly anisotropic electrical and opto-electronic conductance. Moreover, as the thickness of the Pb–I layer increases, the anisotropy is less pronounced, and the rise time and recover time of photo response decrease. Finally, the layer-edge device is demonstrated to be extremely sensitive to environmental humidity, due to the abundant hydroxyl groups which can help water condensation.

## Results

### Sample preparation and layer-edge device fabrication

First, in order to fabricate the layer-edge device, 2D perovskite single crystal with large enough layer-edge surface is required. However, in the commonly studied 2D perovskites, because the Van der Waals interaction between the organic layers is quite weak, an extreme preference of growing along the Pb–I plane leads to a limited thickness of single crystals. In order to increase the growth preference perpendicular to the Pb–I plane, we used large polar organics, HOC_2_H_4_NH_3_^+^ (EA), to form the organic layers so that dipole–dipole force can largely enhance the interaction between organic layers^17,18^. By a cooling method (see Methods), centimeter-wide and millimeter-thick single crystals of (HOC_2_H_4_NH_3_)_2_PbI_4_ were successfully synthesized (Supplementary Figure 1). The lattice structure of the 2D_EA perovskites is confirmed by single-crystal X-ray diffraction (SCXRD), and the layer-edge surfaces are determined (see Methods and Supplementary Figure 2).

**Fig. 1:**
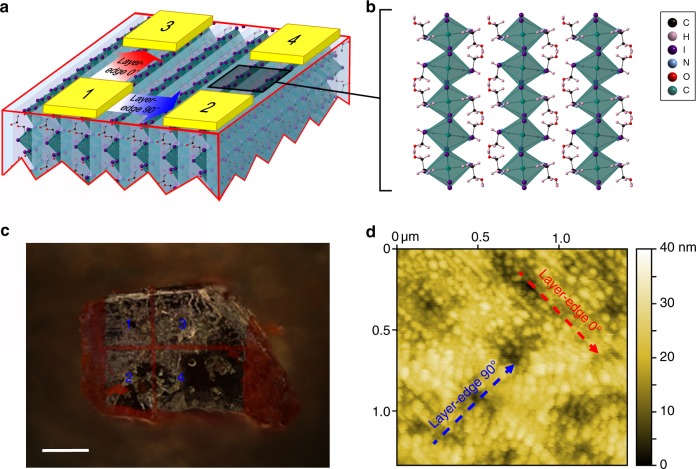
Layer-edge device. **a** Schematic diagram of layer-edge surface of 2D perovskite (HOC_2_H_4_NH_3_)_2_PbI_4_. Blue arrow and red arrow between electrodes indicate the direction of layer-edge 90° and 0°, respectively. **b** Top view of layer-edge surface. **c** Optical image of layer-edge surface device. The scale bar is 400 μm. **d** AFM image of layer-edge surface

For the purpose of investigating the electronic and opto-electronic transport properties of layer-edge surface of 2D perovskites, both layer-edge 0° and layer-edge 90° metal–semiconductor–metal devices are fabricated by depositing Au electrodes with the same active area and deposition conditions (schematically shown in Fig. 1a). The top view of the conducting channel of layer-edge device is schematically shown in Fig. 1b. The optical image of the layer-edge surface device is shown in Fig. 1c, in which the electrode pairs of 1 to 3 and 2 to 4 are layer-edge 0° device, while the electrode pairs of 1 to 2 and 3 to 4 indicate layer-edge 90° device. Device on the in-plane surface was also fabricated for comparative experiments (Supplementary Figure 3). From the AFM data, we can get that the roughness of the layer-edge surface and in-plane surface is about 5 nm (Fig. 1d) and 1 nm (Supplementary Figure 4). The contact resistance is much smaller than the intrinsic resistance of sample, so the contact resistance in our transport measurements is neglected (see Methods). Moreover, the corrections according to the geometry difference of channels in the layer-edge 90° and 0° devices are considered (see Methods).

### *I–V* characterization of layer-edge device

**Fig. 2:**
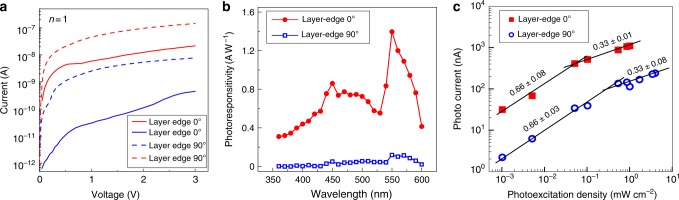
Transport properties of layer-edge device with *n* = 1. **a** *I–V* curve in dark and photo environment under white light with the power of 1 mW cm^−2^. *I*_d_ is dark current and *I*_p_ is photo current for layer-edge 0° and 90° devices. **b** Photo responsivity vs wavelength. The light power density is 10 mW cm^−2^. **c** Photo current vs photoexcitation density in layer-edge 0° and 90° devices. The bias voltage is fixed at 3 V in **b** and **c**

The *I–V* curve (Fig. 2a) shows that the dark current in layer-edge 0° device is about two-orders larger than that in layer-edge 90° device, while the photo current of layer-edge 0° and layer-edge 90° device show about one-order difference. In the photo responsivity measurements, we fixed the bias at 3 volts and measured the photo responsivity over a wide range of incident light wavelength from 360 to 600 nm (Fig. 2b), indicating that layer-edge 0° and 90° devices have the similar wavelength dependence, and the largest photo responsivity was found at 550 nm wavelength. Meanwhile, the magnitude of photo responsivities in the layer-edge 0° device is close to that in the in-plane device (Supplementary Figure 5), and about one-order larger than that in the layer-edge 90° device (Fig. 2b). Such anisotropy of transport properties is similar as the anisotropy observed in the excitonic response in 2D perovskites^19^, indicating that carrier transport mainly occurs along Pb–I–Pb inorganic pathways while is blocked between different inorganic layers owing to confinement by the organic layers, as shown schematically in Fig. 1a. Our study illustrates that in 2D perovskite thin film device, random orientation of crystalline domains will create massive obstacles for carrier transport, hindering the photovoltaic application and fundamental research.

The illumination power density dependent photo current was measured at 3 volts bias voltage and the 550 nm wavelength of incident light, so that the photo responsivity has maximum value. As shown in Fig. 2c, the photo current *I*_p_ and photoexcitation density *D* has the power law relations *I*_p_ ∝ *D*^*m*^. Both layer-edge 0° and layer-edge 90° devices show two distinct power law regions: when the photoexcitation density is low, the trap-assisted recombination dominates with $m = \frac{2}{3}$; as the photoexcitation density is increased, the bimolecular recombination becomes the dominating mechanism, ${\mathrm{leading}}\,{\mathrm{to}}\,m = \frac{1}{3}$. The decrease of *m* compared to the idea value of 1/2 and 1 for the two recombination regimes in 2D perovskite in-plane device has been reported by other groups^20^, where two possible reasons were discussed: (1) exciton–exciton interactions which is largely enhanced in low-dimensional systems, and (2) limited collection of photo-generated charges blocked by insulating organic layers. In the layer-edge device, the many-body interactions still have a key role, while the blockade of charge collection by insulating layers does not exist. Another possible reason of decreased *m* in layer-edge device is proposed here: when the charge carriers are generated at surface, the carriers can diffuse into the bulk before they recombine; as the incident light density increases, the carrier diffusion is suppressed due to carrier recombination, leading to a decrease of carrier lifetime, and finally result in an effective desensitization of photo conductivity^21^.

**Fig. 3:**
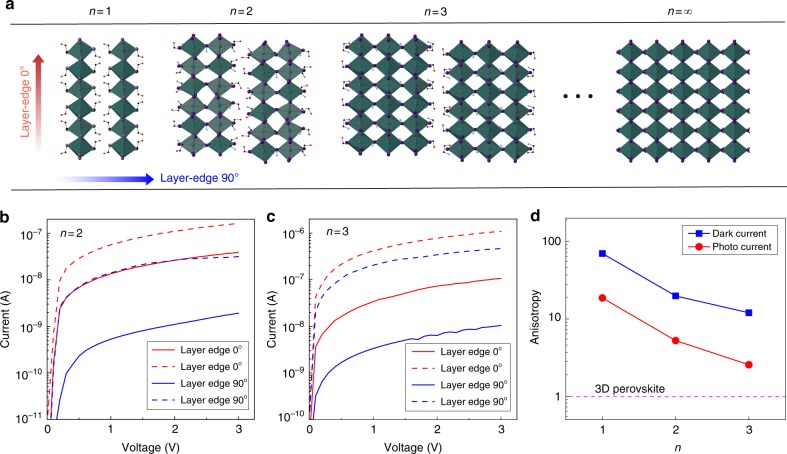
Thickness-dependent electronic/opto-electronic properties. **a** Schematic image of 2D perovskites with *n* = 1, 2, 3, and ∞. Dark and photo current of 2D perovskite with **b** *n* = 2 and **c** *n* = 3. **d** Anisotropy of conductance and photo conductance with different *n*

We also succeeded to grow 2D perovskites with different thickness of inorganic layers. Here we introduce the chemical formula EA_2_MA_*n*−1_PbI_3*n*+1_, where EA = HOC_2_H_4_NH_3_^+^ and MA = CH_3_NH_3_^+^, and *n* represents the number of inorganic layers between two ammonium organic layers consisting of EA. The single-crystal structure of EA_2_MA_*n*−1_PbI_3*n*+1_ for *n* = 1, 2, and 3 are determined by Single Crystal XRD, and the positions of missing organic groups are decided by optimization at GGA/PBE level of theory, as shown in Fig. 3a. Basically, enlarging the thickness of the semiconductor layer in a quantum well structure will decrease the quantum confinement^22–24^. In order to investigate the effect of quantum confinement on the transport properties of layer-edge surface device, the *I–V* curve under dark and photo environment for 2D perovskites with *n* = 2 and 3 were measured, as shown in Fig. 3b and c. Here we define the anisotropy of conductance and photo conductance as the ratio of current with bias equals to 3 volts in layer-edge 0° device and 90° device. As shown in Fig. 3d, The anisotropy is decreased as *n* is increased; when *n* → *∞* the case of 3D perovskite is approached, and the electronic anisotropy is minimized and equals to one.

Generally, layer-edge 0° and layer-edge 90° devices have different transport mechanism. In layer-edge 0° device, the carrier transport mainly happens within the individual semiconductor layers; when the external electric field is applied parallel to the semiconductor layers, the carriers are drifted until scattered by charge impurities or phonons. So the transport in layer-edge 0° device can perfectly described by Drude model, in which the mobility is determined by effective mass and scattering relaxation time. On the other hand, in layer-edge 90° device, the dominating process of transport is tunnelling through organic barriers, and Drude model is invalid. Actually, when we estimated the effective mass from the DFT calculated band structures (Supplementary Figure 6), the effective masses along semiconductor plane (the situation in layer-edge 0° device) are similar for *n* = 1, 2, and 3; While the effective mass perpendicular to the semiconductor plane is smallest for *n* = 1, and close to infinite for *n* = 2 and 3, as shown in Supplementary Table 1. Those effective masses cannot explain the *n* dependent anisotropy of conductivity in the sense of Drude model, confirming that Drude model cannot be used for layer-edge 90° device.

### Photo response of layer-edge device

**Fig. 4:**
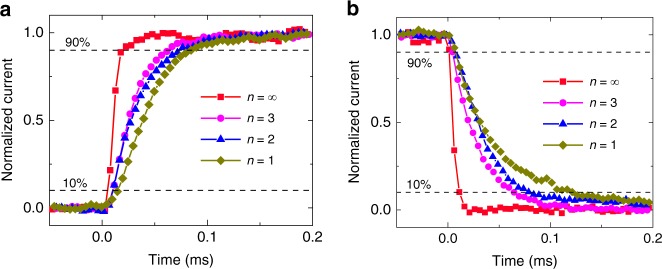
Thickness-dependent photo response. **a** Photo response and **b** recovery of normalized current in 2D perovskite with *n* = 1, 2, and 3 and 3D perovskite

To study the photo-response dynamics in layer-edge surface devices, we measured the time-dependent photo current of 2D perovskites (*n* = 1, 2, 3) and 3D perovskites. The timescales of photo-response dynamics measurement are quite larger than the carrier of lifetime in regular time-resolved photoluminescence measurement, since the external electric field will dissemble the electron–hole pairs in the excitons, and effectively extend the carrier lifetime^25^. The response time *τ* is defined by the duration of photo current changing from 10 to 90% of the maximum and vice versa. The rise and recover current curves are shown in Fig. 4a and b, respectively, and the response time *τ* is decreased with the increasing layer number *n* and reaches the minimum value as *n* = ∞ (3D perovskites), as shown in Table 1. The tendency of decrescent *τ* is highly associated with the quantum confinement effect, originated from the organic–inorganic–organic sandwich structures in 2D perovskites: as *n* increases, the quantum confinement effect is weakened, resulting in the increase of carrier mobility^22^ that shortens the drift time of photo-generated carriers to the electrodes, and finally making the *τ* decrease.

**Table 1:** Rise and recover time of photo response measurement

	*n* = 1	*n* = 2	*n* = 3	*n* = ∞
Rise time (μs)	69.8	63.9	54.4	15.0
Recover time (μs)	106.9	74.2	50.0	10.3

### Humidity sensing of layer-edge device

**Fig. 5:**
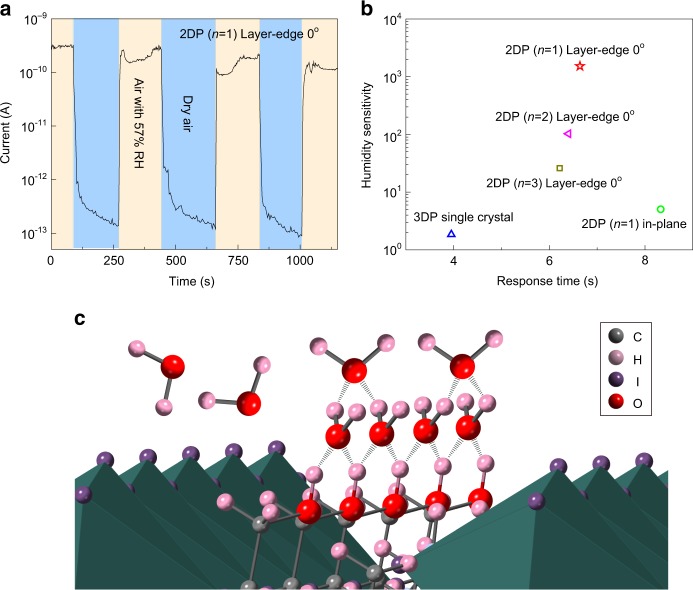
Humidity sensitivity. **a** Dark current in dry air and with 57% humidity air in 2D perovskite layer-edge 0° device. **b** Humidity sensitivity and response time for devices on layer-edge 0° surface with different *n*, in-plane surface in 2D perovskites with *n* = 1, and (110) surface in 3D perovskites. **c** Schematic diagram of water absorption in layer-edge surface of 2D perovskites

Since the surface of perovskite device is significantly influenced by the environment gases and humidity, the transport behavior, which mainly occurs near the surface of the single-crystal device, are quite sensitive to the humidity when the device is operated in the air. In 3D perovskites, the sensitivity of humidity have been studied in the single crystal^26^ and thin film^27^. However, the result in 2D perovskites is expected to be quite different because of its layered structure and different organic groups adopted, especially in the layer-edge device some abnormal humidity sensitivity may show up. As shown in Fig. 5a, the currents of layer-edge 0° devices (with *n* = 1) in dry air and 57% relative humidity (RH) air are measured (see Methods), showing reversible three-order-magnitude change, and response time to the water absorption as fast as approximately 6.6 s. Here the response time is defined as the time required to change the current amplitude at dry air to the 90% maximum at 57% RH. The humidity response for layer-edge device with *n* = 2 and 3 (Supplementary Figure 7), layer-edge device 90° and in-plane device in 2D perovskite with *n* = 1, and (110) surface device in 3D perovskite were also measured (Supplementary Figure 8). We estimate the performance of humidity sensing for each perovskite device by the humidity sensitivity (dividing the current in 57% RH by the current in dry air), and the response time, as shown in Fig. 5b. The 2D perovskite layer-edge 0° device with *n* = 1 presents the best humidity sensitivity, and relative outstanding response time. Especially compared to the best humidity sensor based on hybrid perovskite so far^28^, the performance of humidity sensitivity and response time in 2D perovskite layer-edge device with *n* = 1 are much more excellent. As *n* increases, the humidity sensitivity of layer-edge device is decreased. The reason is that the ratio of organic layers to the inorganic layers will decrease as *n* increases, which means that the density of hydroxyl groups on the layer-edge surface decreases, and the ability to absorb water decreases as well.

Now we try to explain such marked high humidity sensitivity of layer-edge device. First of all, the layer-edge surface of 2D perovskite is very rough as shown in the AFM image (Fig. 1d), especially compared to the in-plane surface (after mechanical exfoliation) in which the AFM shows atomic flat surface with much less defects (Supplementary Figure 4). Such structural defects in layer-edge surface can trap a lot of water molecules, which is proved by the large difference between the humidity sensitivity of layer-edge surface device and in-plane surface device (Supplementary Figure 8b). On the other hand, the layer-edge surface has abundant hydroxyl groups, and is easy to absorb the water in the air (as shown schematically in Fig. 5c). This mechanism can be verified when we look at the speed of current recovery when the device is put back to dry air from 57% humidity air. The current recovery speed in 2D perovskite with hydroxyl groups (Fig. 5a) is much slower than that in 2D perovskite (C_6_H_5_(CH_2_)_2_NH_3_)_2_PbI_4_ (2D_PEA perovskites) in which hydroxyl groups are absent (Supplementary Figure 9), indicating a process of desorbing water from hydroxyl groups. The type of moving charges in the layer-edge surface device in humidity environment is still unknown. The electronic carriers might contribute to the high humidity sensitivity since the doping water may change the surface energy band structures^29^, or increase the carrier (electron or hole) concentration. On the other hand, when the water is condensed at the layer-edge surface, the water molecules may hydrolyze the perovskite lattices^30^, and create huge amounts of moving ions. Finally, protons can also transfer between the water molecules, which is called Grotthuss mechanism^31,32^.

## Discussion

In summary, we succeed to fabricate the layer-edge surface device of 2D hybrid perovskites with different inorganic layer thickness, and investigate their transport properties. Strong anisotropic electronic/opto-electronic properties are demonstrated, caused by the organic/inorganic layer staggered multiple quantum well structures. Such anisotropy is decreased as the thickness of inorganic layer increases. The existence of large anisotropy of electronic transport in 2D perovskites indicates the importance of the choice of crystalline orientation in the layer-edge device. We also obtain extremely high humidity sensitivity in the layer-edge device due to the abundant hydroxyl groups. The humidity sensing properties of layer-edge device in 2D perovskite strongly depend on the organic groups adopted, indicating urgent requirement of research on 2D perovskites with different organic layers in the future. Our study has revealed the unusual transport properties of layer-edge surface, and indicated potential applications of humidity sensor based on 2D perovskite single crystals.

## Methods

### Synthesis of perovskite single crystals

2D perovskites (HOC_2_H_4_NH_3_) single crystals are grown using a cooling method. First, 1.6 g PbI_2_ is put in 16 ml of 57% HI solution in a sample vial, and dissolved by sonication at room temperature. Thereafter, 0.8 ml HOC_2_H_4_NH_2_ was add and the precipitate. The solution is then cooled down to 4 °C. After one week, the crystal was taken out and put in a fresh prepared solution. Such process was repeated for 5 weeks.

In order to grow *n* = 2 perovskites, we prepare 16 ml HI (57 wt. %) and put in 5.5 g PbI_2_ powder. After the powder dissolved, add 0.4 ml MA solvent and 0.8 ml EA solvent, then heat it to 90 °C. After 1 h, cool the solvent to 60 °C and keep it for 2 days, then cool it down to room temperature. Then large size single crystal will be synthesized in a few days. For *n* = 3 perovskite single crystal, we prepare 12 ml HI solution, we need to add 5 g PbI_2_ powder, 0.4 ml MA and 0.8 ml EA. Then the heating and cooling process is the same as *n* = 2 perovskites.

### Single crystal XRD of 2D perovskite

Single-crystal intensity data were collected using a Bruker APEX DUO with Mo *K*_α_ radiation (wavelength 0.71073 Å) at 100 K. The cif file is upload to CCDC database, as shown in Data availability. The crystallographic plane of the large single crystal was probed using a Bruker D8 Venture SCXRD Cu target.

### Determine the surface of single crystal

As shown in Supplementary Figure 1, all the single crystals have a shape of thin plate, and the largest surface is defined as in-plane surface. The in-plane surface is parallel to the quantum well superlattice plane, which is proved by the XRD on the in-plane surface, as shown in Supplementary Figure 2a. The layer-edge surface is perpendicular to the in-plane surface as shown in Supplementary Figure 2b, so we can confirm that the layer-edge surface is perpendicular to the superlattice plane.

### Electrical and opto-electronic characterization/optical testing

Electrical properties were measured using a Keithley 4200-SCS parameter analyzer with a Keithley 4210-PA. A Newport 1000 W Xenon Lamp 66921 light source with a Newport monochromator was used for the photo-responsivity measurements. To confirm the size and uniform intensity distribution of the incident light spot, the optical profile was measured by a beam profiling camera (Ophir Spiricon silicon CCD camera SP620U) and the power at each wavelength was measured using a calibrated photodiode (Newport 818-UV). The photo responsivity was calculated using the following formula:

1 $${\mathrm{Photoresponsivity}} = (I_{\mathrm{p}} - I_{\mathrm{d}})/D$$

where *I*_p_ and *I*_d_ are the currents under photoexcitation and in the dark, respectively, and *D* is the illumination power in active channel area in the device.

The setup of photo-response measurement is shown in Supplementary Figure 10.

The setup of current measurement with various atmosphere ambient is shown in Supplementary Figure 11.

### Contact resistance

As shown in Supplementary Figure 12a, we apply a voltage bias (*V*_b_) on the electrode 2 to 3, and measure the current (*I*). The measured resistance between electrodes 2 to 3 include the intrinsic resistance of crystal (*R*_i_) and contact resistance (*R*_c_) between the crystal and the electrodes, i.e., *R*_i_ + *R*_c_ = *V*_b_/*I*_2–3_.

Then, as shown in Supplementary Figure 12b, the voltage bias is applied between the electrode 1 to 4, and we measure the current *I* and the voltage drop *V*_b_ between electrodes 2 to 3 at the same time. Then we can exclude the contact resistance and only measure the intrinsic resistance between electrode 2 to 3: *R*_i_ = *V*_2–3_/*I*_1–4_.

Finally, we find that *V*_b_/*I*_2–3_ ≈ *V*_2–3_/*I*_1–4_, so *R*_i_ + *R*_c_ ≈ *R*_i_. As a result, we can neglect the contact resistance for the *I*–*V* measurement.

### Different device geometry

Also, the channel length between two electrodes is kept at 50 μm; while we try our best to make the channel width *l* close to 500 μm. But sometimes, the geometry may be different for different devices. In order to eliminate the effect of different channel geometry when we calculate the anisotropy of resistance, we do some corrections:

$${\mathrm{Anisotropy = }}\left( {I_{{\mathrm{layer - edge}}\,{\mathrm{0}}^ \circ }/I_{{\mathrm{layer - edge}}\,{\mathrm{90}}^ \circ }} \right) \times \left( {I_{{\mathrm{layer - edge}}\,{\mathrm{90}}^ \circ }/I_{{\mathrm{layer - edge}}\,{\mathrm{0}}^ \circ }} \right)$$

Where *I* is the current, and *l* is the channel width.

## Electronic supplementary material

Supplementary Information

## Data Availability

The data that support the findings of this study are available from the corresponding author upon reasonable request. The X-ray crystallographic coordinates for the structure of 2D perovskites has been deposited at the Cambridge Crystallographic Data Centre (CCDC), under deposition number 1869720 for (HOCH_2_CH_2_NH_3_)_2_PbI_4_. These data can be obtained free of charge from The Cambridge Crystallographic Data Centre via www.ccdc.cam.ac.uk/data_request/cif.
