# Supplementary material for: Layer-edge device of two-dimensional hybrid perovskites
Source: Nat Commun. 2018 Dec 5;9:5196. doi: 10.1038/s41467-018-07656-2 (PMC6281625; doi:10.1038/s41467-018-07656-2)
Supplement: Supplementary file 1 — Supplementary Information [file 41467_2018_7656_MOESM1_ESM.pdf]

Supplementary Information for

Layer-Edge Device of 2D Hybrid Perovskites

Bin Cheng et al.

Supplementary Figures:

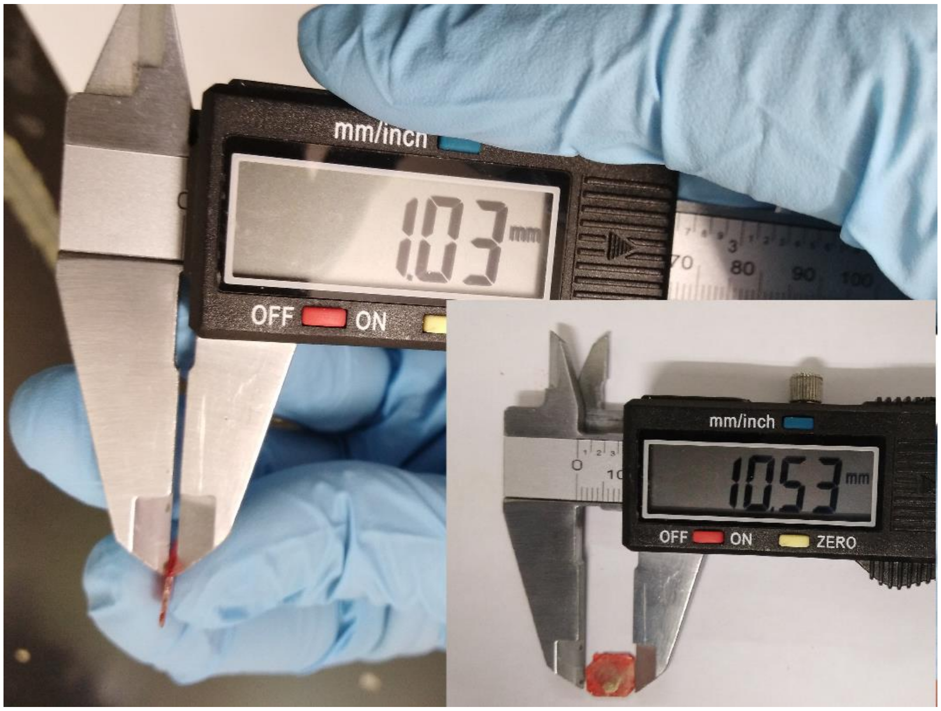

Supplementary Figure 1 | Optical image of centimeter-size and millimeter-thick 2D perovskite single crystal.

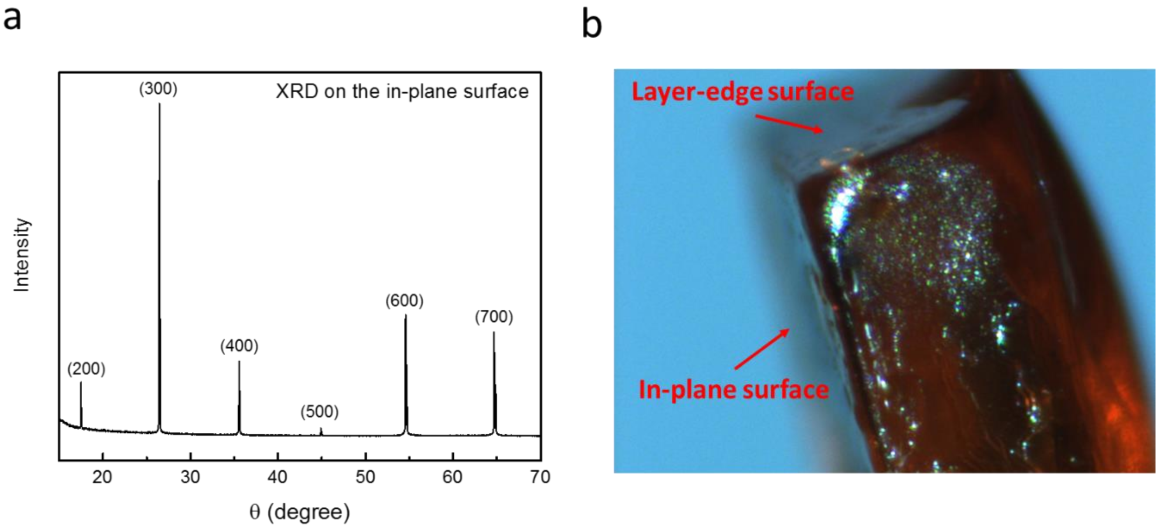

Supplementary Figure 2| a, XRD data on in-plane surface. b, optical image of in-plane surface and layer-edge surface. The angle between the in-plane and layer-edge surface are about 90° ± 3°.

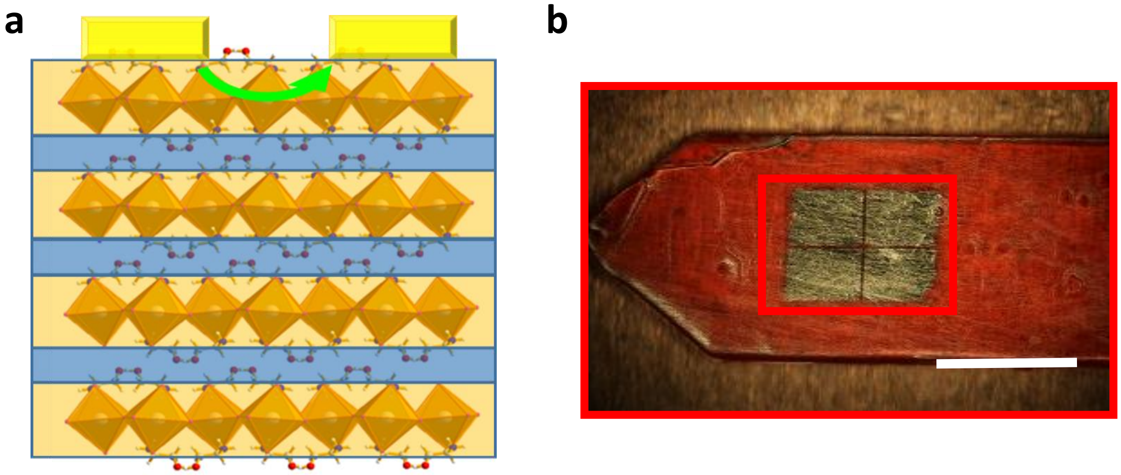

Supplementary Figure 3 | Geometry of in-plane Device. a, Schematic figure and **b,** photo of in-plane device in 2D perovskites**.** The active channel length is 20um, which is made by shadow mask when the e-beam evaporator is used. Scale bar: 500 um.

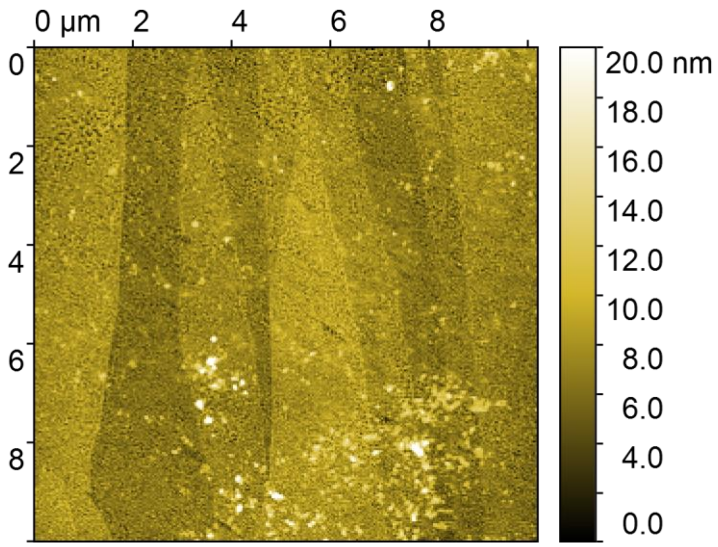

Supplementary Figure 4 | AFM image of in-plane surface in 2D perovskites.

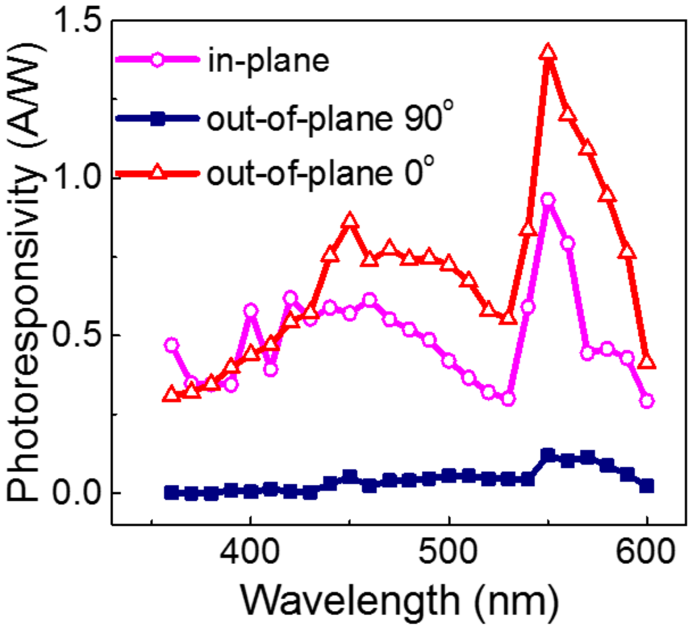

Supplementary Figure 5 | Photoresponsivity of in-plane device and layer-edge device in 2D perovskites.

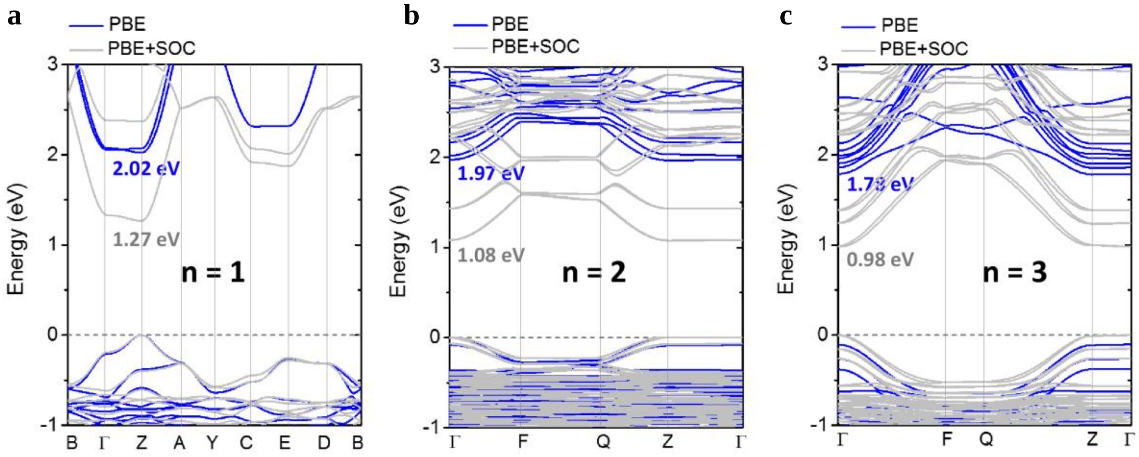

Supplementary Figure 6 | a, Calculated band structures of 2D perovskites at GGA/PBE level with and without account of SOC for *n* = 1, 2, 3. **b,** Thickness dependent photoluminescence.

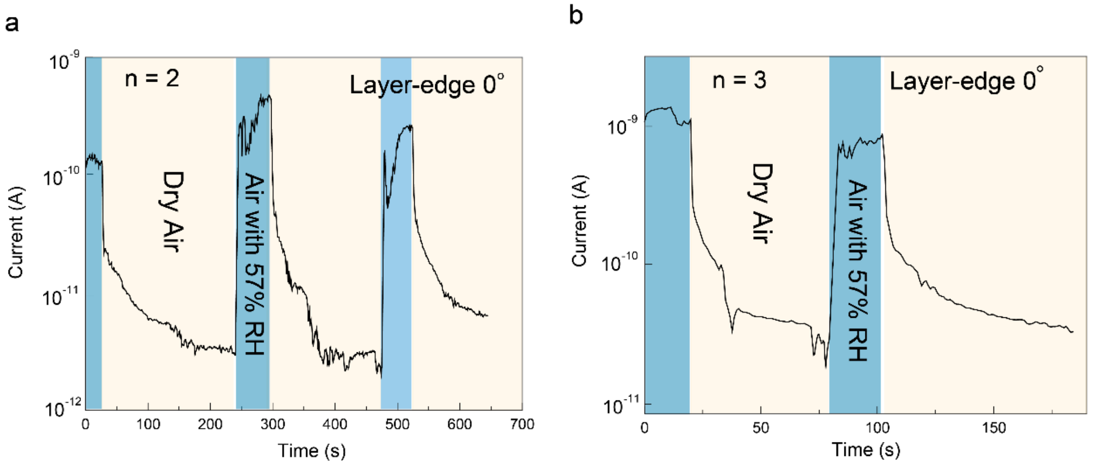

Supplementary Figure 7 | Humidity response of layer-edge surface device with **a,** $n = 2$ and **b,** $n = 3$.

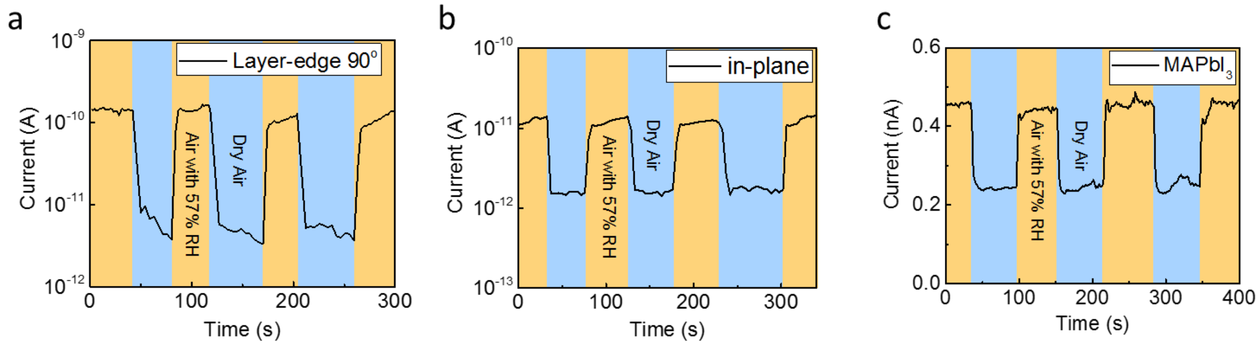

Supplementary Figure 8 | Humidity response of a, layer-edge 90° device in 2D perovskite, **b**, in-plane device in 2D perovskite and **c**, (110) surface of $MAPbI_3$ (3D perovskite).

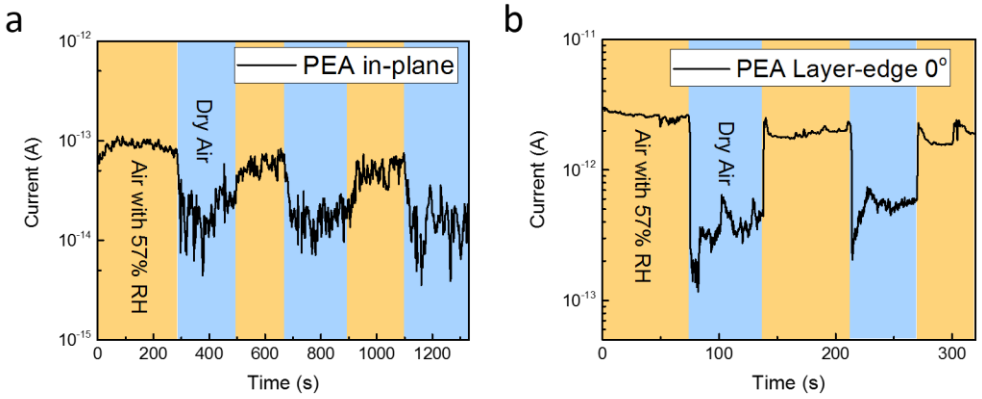

Supplementary Figure 9 | Humidity response of a, in-plane device in 2D PEA perovskite, **b**, layer-edge 0° device in 2D PEA perovskite

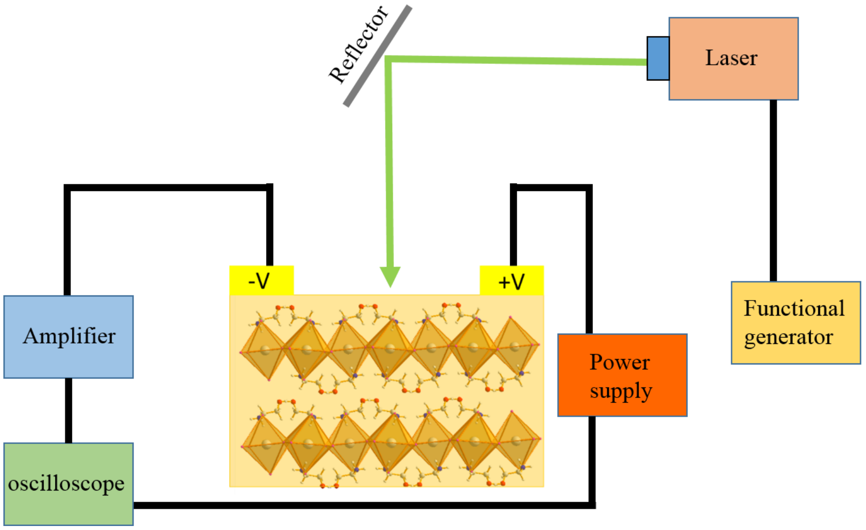

Supplementary Figure 10 | Setup of photo-response measurement. The Power supply is using Keithley 4200SCS, the oscilloscope is using Agilent DSO 5034A, the amplifier is using SRS SR570, the 520 nm laser is using, the pulse function generator is using Agilent 81150A.

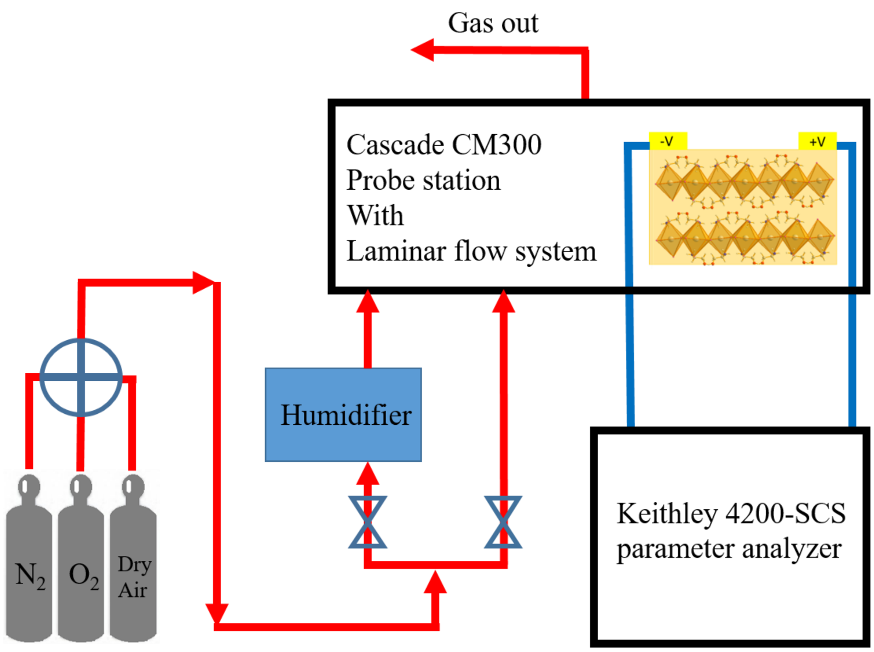

Supplementary Figure 11 | Setup of current measurement with various atmosphere ambient. The setup can immediately switch various atmosphere ambient making the data more precious.

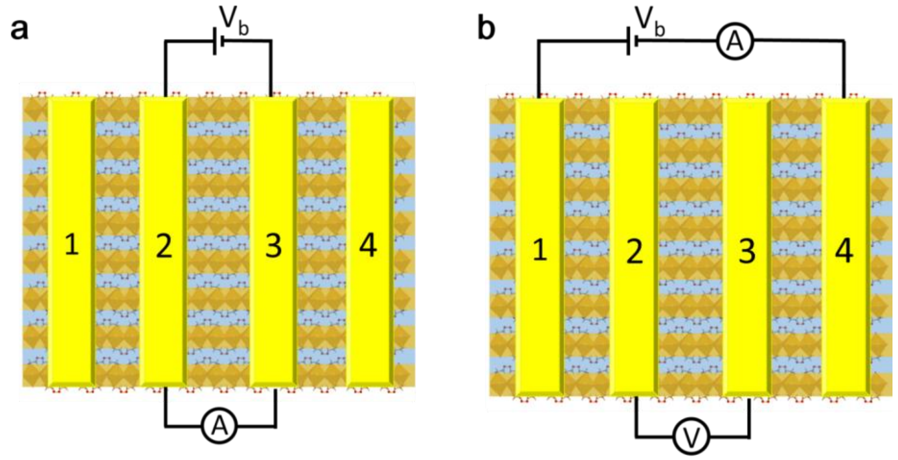

Supplementary Figure 12 | Measurement setup for contact resistance.

Supplementary Tables:

Supplementary Table 1: Effective mass of 2D perovskite with *n* = 1, 2 and 3.

effective mass (×m_0)	***n* = 1**	***n* = 2**	***n* = 3**
Electron	$m_{//}$: 0.223<br>$m_{\perp}$: 2.037	$m_{//}$: 0.181<br>$m_{\perp}$: close to infinite	$m_{//}$: 0.239<br>$m_{\perp}$: close to infinite
Hole	$m_{//}$: 0.509<br>$m_{\perp}$: 0.665	$m_{//}$: 0.264<br>$m_{\perp}$: close to infinite	$m_{//}$: 0.302<br>$m_{\perp}$: close to infinite
